# Room-temperature X-ray crystallography reveals the oxidation and reactivity of cysteine residues in SARS-CoV-2 3CL M^pro^: insights into enzyme mechanism and drug design

**DOI:** 10.1107/S2052252520012634

**Published:** 2020-09-21

**Authors:** Daniel W. Kneller, Gwyndalyn Phillips, Hugh M. O’Neill, Kemin Tan, Andrzej Joachimiak, Leighton Coates, Andrey Kovalevsky

**Affiliations:** a Neutron Scattering Division, Oak Ridge National Laboratory, 1 Bethel Valley Road, Oak Ridge, TN 37831, USA; b Center for Structural Genomics of Infectious Diseases, Consortium for Advanced Science and Engineering, University of Chicago, Chicago, IL 60667, USA; c Structural Biology Center, X-ray Science Division, Argonne National Laboratory, Argonne, IL 60439, USA; d Department of Biochemistry and Molecular Biology, University of Chicago, Chicago, IL 60367, USA

**Keywords:** room-temperature X-ray crystallography, SARS-CoV-2, 3CL main protease, 3CL M^pro^, cysteine oxidation, protonation state, enzyme mechanism, drug design

## Abstract

The emergence of the novel coronavirus SARS-CoV-2 has resulted in a worldwide pandemic not seen in generations. Creating treatments and vaccines to battle COVID-19, the disease caused by the virus, is of paramount importance in order to stop its spread and save lives. The viral main protease, 3CL M^pro^, is indispensable for the replication of SARS-CoV-2 and is therefore an important target for the design of specific protease inhibitors. Detailed knowledge of the structure and function of 3CL M^pro^ is crucial to guide structure-aided and computational drug-design efforts. Here, the oxidation and reactivity of the cysteine residues of the protease are reported using room-temperature X-ray crystallography, revealing that the catalytic Cys145 can be trapped in the peroxysulfenic acid oxidation state at physiological pH, while the other surface cysteines remain reduced. Only Cys145 and Cys156 react with the alkylating agent *N*-ethylmaleimide. It is suggested that the zwitterionic Cys145–His45 catalytic dyad is the reactive species that initiates catalysis, rather than Cys145-to-His41 proton transfer via the general acid–base mechanism upon substrate binding. The structures also provide insight into the design of improved 3CL M^pro^ inhibitors.

## Abstract 2

The viral main protease is indispensable for SARS-CoV-2 replication, and detailed knowledge of its structure and function is crucial to guide structure-aided and computational drug-design efforts. X-ray crystallography is used to reveal the oxidation and reactivity of the cysteine residues of the protease, providing insights into the enzyme mechanism and drug design.

## 1. Introduction   

The coronavirus-induced disease COVID-19 has swiftly spread to every corner of the globe, causing a severe pandemic not seen in a century. With over 30 million confirmed cases worldwide to date and no approved treatments or vaccines, COVID-19, which is caused by the novel coronavirus SARS-CoV-2 (Severe acute respiratory syndrome coronavirus 2; Helmy *et al.*, 2020 ▸; Wu *et al.*, 2020 ▸; Corona­viridae Study Group of the International Committee on Taxonomy of Viruses, 2020 ▸; Liu *et al.*, 2020 ▸), has resulted in almost every country taking unprecedented social measures to limit its spread. SARS-CoV-2, a single-stranded enveloped RNA virus, is related to the common cold viruses, and its ∼30 kb genome is 82% identical to that of the SARS coronavirus (SARS-CoV) that was responsible for an outbreak in 2003 (Xu *et al.*, 2020 ▸). However, SARS-CoV-2 appears to be more contagious than similar viruses. The replication of such viruses is fully dependent on the function of a 3-chymotrypsin-like protease enzyme (3CL M^pro^) that hydrolyzes large polyproteins to generate essential nonstructural proteins, which play a fundamental role in transcription/replication during infection (Hilgenfeld, 2014 ▸; Gorbalenya & Snijder, 1996 ▸; Muramatsu *et al.*, 2016 ▸). The SARS-CoV-2 3CL M^pro^ enzyme operates at no fewer than 11 cleavage sites on two large polyproteins 1a and 1ab (replicase 1a, ∼450 kDa, and replicase 1ab, ∼790 kDa). As 3CL M^pro^ enzymes are indispensable for viral proliferation and owing to the lack of homologous human proteins, these enzymes are promising targets for the design and development of specific protease inhibitors (Pillaiyar *et al.*, 2016 ▸; Wang *et al.*, 2017 ▸; Dai *et al.*, 2020 ▸; Jo *et al.*, 2020 ▸; Zhang *et al.*, 2020 ▸; Jin, Du *et al.*, 2020 ▸) and the repurposing of existing clinical drugs (Jin, Zhao *et al.*, 2020 ▸; Ma *et al.*, 2020 ▸; Riva *et al.*, 2020 ▸). The catalytically active SARS-CoV-2 3CL M^pro^ enzyme is a dimer. Cleavage of the large polyprotein chains by 3CL M^pro^ occurs at the glutamine residue in the P1 position of the substrate via a Cys145–His41 dyad, in which the cysteine thiol functions as the nucleophile in the proteolytic process (Jin, Zhao *et al.*, 2020 ▸).

**Figure 1:**
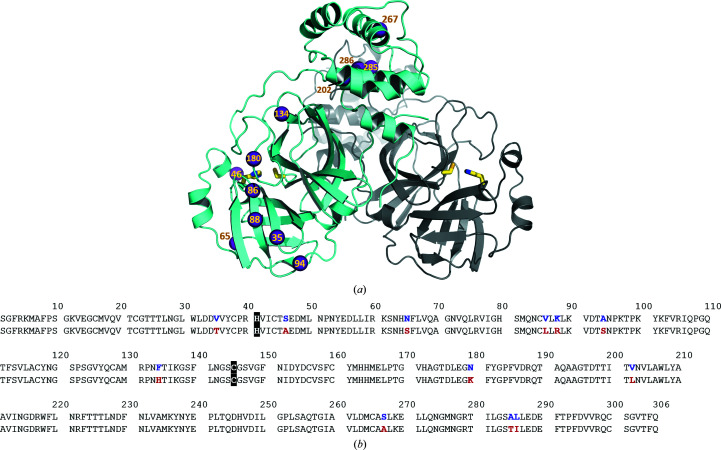
(*a*) Structure of the 3CL M^pro^ dimer from SARS-CoV-2 (PDB entry 6wqf; Kneller *et al.*, 2020 ▸) in cartoon representation. The locations of the 12 amino-acid substitutions relative to the SARS-CoV enzyme are shown as violet spheres in one monomer. The catalytic Cys–His dyad is shown as sticks in each monomer. (*b*) Amino-acid sequence alignment of 3CL M^pro^ from SARS-CoV-2 (first row) and SARS-CoV (second row) indicating the positions of the amino-acid substitutions. The catalytic His41 and Cys145 are highlighted in black.

SARS-CoV-2 3CL M^pro^ has 12 amino-acid substitutions compared with the SARS-CoV homolog that occur throughout the sequence in the catalytic domains I and II, and in the helical domain III (Fig. 1 ▸; Xu *et al.*, 2020 ▸; Jin, Du *et al.*, 2020 ▸; Anand *et al.*, 2003 ▸; Yang *et al.*, 2005 ▸). These substitutions do not appear to significantly affect the function of SARS-CoV-2 3CL M^pro^, as its catalytic efficiency is very similar to that of the SARS-CoV enzyme (Zhang *et al.*, 2020 ▸; Jin, Du *et al.*, 2020 ▸; Xue *et al.*, 2007 ▸; Huang *et al.*, 2004 ▸; Solowiej *et al.*, 2008 ▸). Therefore, in mechanistic terms, it can be expected that both enzymes catalyze the peptide-hydrolysis reaction in a similar fashion. The exact enzyme mechanism, however, is still unknown and is poorly characterized. Specifically, it has been debated whether substrate hydrolysis is initiated by proton transfer from the thiol of Cys145 to the imidazole of His41 within the catalytic Cys–His dyad through a general acid–base mechanism (Huang *et al.*, 2004 ▸) to give a deprotonated negatively charged thiolate on Cys145 and a protonated positively charged imidazolium on His41, or whether this ion pair (or zwitterion) already exists before substrate binding (Solowiej *et al.*, 2008 ▸; Paasche *et al.*, 2014 ▸). In either case, the formed thiolate (S^−^) would be a much more reactive species (*i.e.* a better nucleophile) towards the C atom of the scissile peptide bond than a protonated thiol (SH) would be. Here, we used room-temperature X-ray crystallography to present experimental evidence that Cys145 in SARS-CoV-2 3CL M^pro^ is highly reactive and can easily be oxidized to an uncommon peroxysulfenic thiol adduct at neutral pH. In contrast, the other 11 Cys residues in the protein sequence are much less prone to oxidation, even though several of them, namely Cys85, Cys156, Cys160 and Cys300, are located on the protein surface and are exposed to the bulk solvent. Remarkably, in our room-temperature X-ray structure of SARS-CoV-2 3CL M^pro^ obtained from a crystal grown at the acidic pH of 6.0, Cys145 is not oxidized. In addition, Cys145 and Cys156 readily react with the alkylating reagent *N*-ethylmaleimide in the crystal. Our crystallographic data obtained at the near-physiological temperature of 293 K and pH values of 7.0 and 6.0 provide relevant information on the catalytic mechanism of 3CL M^pro^, the function of the enzyme and the design of specific protease inhibitors to battle COVID-19.

## 2. Materials and methods   

### 2.1. General information   

Protein-purification supplies were purchased from GE Healthcare, Piscataway, New Jersey, USA. Crystallization reagents were purchased from Hampton Research, Aliso Viejo, California, USA.

### 2.2. Cloning, expression and purification of 3CL M^pro^   

The 3CL M^pro^ (Nsp5 M^pro^) from SARS CoV-2 was cloned into the pMCSG81-Delta238 plasmid, designated pCSGID-Mpro, expressed and purified according to the published procedure (Kneller *et al.*, 2020 ▸). To generate the authentic protease, the N-terminus is flanked by the maltose-binding protein followed by the 3CL M^pro^ autocleavage site SAVLQ↓SGFRK (the arrow indicates the cleavage site) corresponding to cleavage between NSP4 and NSP5 in the viral polyprotein. At the C-terminus, the construct codes for the Human rhinovirus 3C PreScission protease cleavage site (SGVTFQ↓GP) connected to a His_6_ tag. The N-terminal flanking sequence is autoprocessed by 3CL M^pro^ during expression, whereas the C-terminal flanking sequence is removed by treatment with PreScission protease. For crystallization, the authentic 3CL M^pro^ was concentrated to 5 mg ml^−1^.

### 2.3. Crystallization   

The protein was first crystallized in 0.1 *M* bis-Tris pH 6.5, 22% PEG 3350. Several crystal ‘flower’ aggregates were collected from this condition and were used to generate microseeds using a Hampton Research Seed Bead kit. For room-temperature X-ray crystallography, crystals were grown in 10 µl sitting drops made by mixing the protein sample (5 mg ml^−1^) and reservoir solution (0.1 *M* bis-Tris pH 6.5, 18–20% PEG 3350) in a 1:1 ratio with 0.2 µl of microseeds (1:200 dilution). This condition was formulated to produce a pH of 7.0 in the crystallization drop, which was confirmed by direct pH measurement using a microelectrode. Single plate-like crystals grew in several days and continued to grow at 14°C. To obtain fresh crystals with a native (non-oxidized) Cys145, a batch setup was used to grow crystals in 200 µl drops containing protein at 6.6 mg ml^−1^ in 10% PEG 3350, 0.1 *M* bis-Tris pH 7.0 with 0.4 µl of microseeds.

### 2.4. Generation of structures **I**–**IV**   

Structure **I** containing a peroxysulfenate-modified Cys145 was obtained by soaking a native 3CL M^pro^ crystal transferred from a crystallization drop containing 0.5 m*M* TCEP in the corresponding crystallization well solution that contained no reducing agent for one hour at 14°C. Structure **II** containing a peroxysulfenate-modified Cys145 and an *N*-ethylmaleimide-modified Cys156 was obtained by first manipulating another crystal in the same fashion, producing structure **I**, but was then soaked in the dark for 72 h at 4°C in a solution containing 2 m*M* *N*-ethylmaleimide originally dissolved in 100% DMSO. Structure **III** containing *N*-ethylmaleimide-modified Cys145 and Cys156 was obtained by soaking a fresh native 3CL M^pro^ crystal in a solution containing 2 m*M* *N*-ethylmaleimide for one hour in the dark at room temperature. Structure **IV** in the monoclinic space group *P*2_1_ containing the native Cys145 was generated by growing crystals in 0.1 *M* bis-Tris pH 6.0, 18–20% PEG 3350 using a protein sample dialyzed into the same buffer to guarantee a pH of 6.0 in the crystallization drops.

### 2.5. X-ray data collection and structure refinement   

To collect room-temperature diffraction data sets, crystals of 3CL M^pro^ were mounted using the MiTeGen (Ithaca, New York, USA) room-temperature capillary setup. Room-temperature X-ray crystallographic data for the crystals producing structures **I**, **II**, **III** and **IV** were collected immediately following soaking using a Rigaku HighFlux HomeLab instrument equipped with a MicroMax-007 HF X-ray generator and Osmic VariMax optics. The diffraction images were obtained using an EIGER R 4M hybrid photon-counting detector. The diffraction data were integrated using the *Crys­Alis^Pro^* software suite (Rigaku, The Woodlands, Texas, USA). The diffraction data were then reduced and scaled using *AIMLESS* (Evans & Murshudov, 2013 ▸) from the *CCP*4 suite (Winn *et al.*, 2011 ▸). Molecular replacement using PDB entry 6wqf (Kneller *et al.*, 2020 ▸) was then performed with *MOLREP* (Vagin & Teplyakov, 2010 ▸) from the *CCP*4 suite. Refinement of each protein structure was conducted using *phenix.refine* from the *Phenix* suite of programs (Liebschner *et al.*, 2019 ▸) and the *Coot* molecular-graphics program (Emsley *et al.*, 2010 ▸). The geometry of each final structure was then carefully checked with *MolProbity* (Chen *et al.*, 2010 ▸). The data-collection and refinement statistics are given in Supplementary Table S1.

## 3. Results   

### 3.1. The Cys145 thiol is readily oxidized at pH 7   

**Figure 2:**
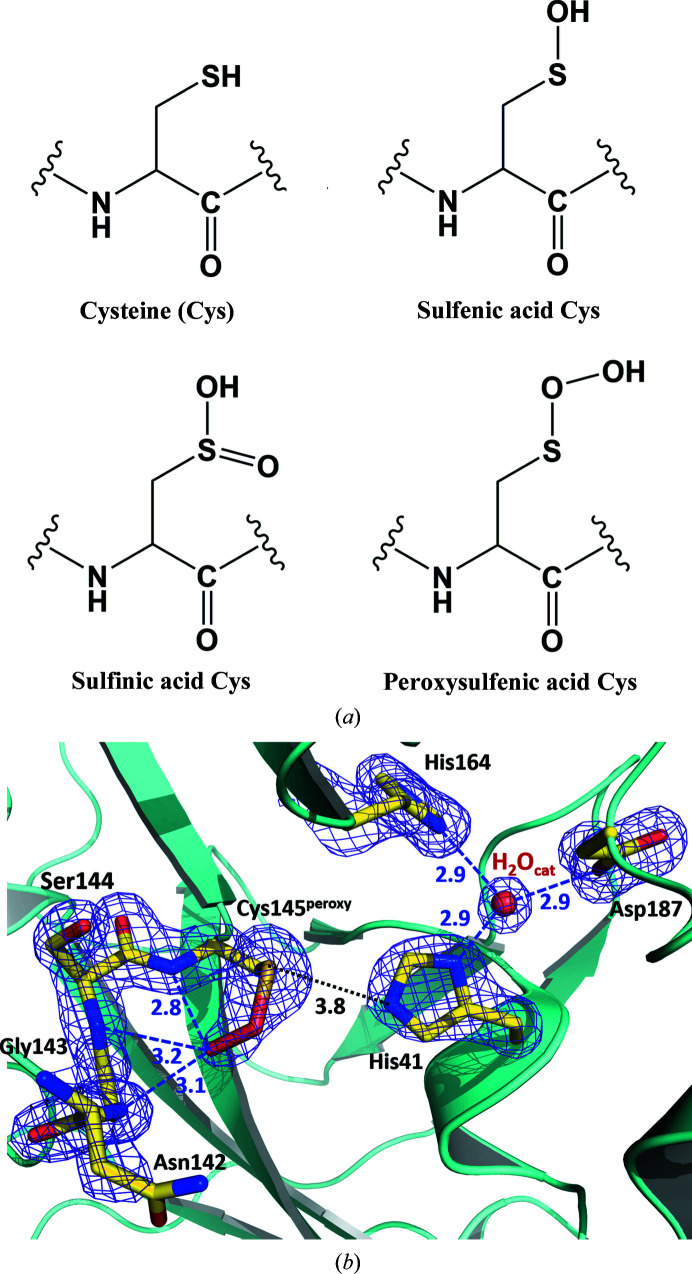
(*a*) Chemical diagrams of possible oxidation states of a cysteine side chain. (*b*) The catalytic site of SARS-CoV-2 3CL M^pro^ structure **I**. Possible hydrogen bonds are shown as blue dashed lines; the distance between Cys145 and His41, which is too long for a hydrogen bond, is shown as a black dotted line. The 2*F*_o_ − *F*_c_ electron-density map contoured at the 1.5σ level is shown as a purple mesh. All distances are given in ångströms.

In our first room-temperature X-ray structure of ligand-free SARS-CoV-2 3CL M^pro^, structure **I**, obtained at 1.80 Å resolution, we observed that Cys145 is in the rarely seen peroxy­sulfenic (—S—O—OH) thiol oxidation state that is presumably formed by direct reaction of the cysteine S atom with the molecular oxygen dissolved in water [Figs. 2 ▸(*a*) and 2 ▸(*b*)]. We obtained this structure using a crystal that grew within three days and was then soaked overnight in the crystallization well solution that contained no reducing agents at pH 7 (Supplementary Figs. S1 and S2). Such fresh crystals normally did not exhibit an oxidized Cys145, as seen in our previously reported room-temperature structure of the ligand-free enzyme (PDB entry 6wqf; Kneller *et al.*, 2020 ▸). Importantly, we could also reproduce structure **I** by letting the enzyme crystals grow for more than one week at 14°C, thus indicating that the 0.5 m*M* TCEP reducing agent present in the crystallization drops does not prevent Cys145 oxidation over a prolonged duration.

It is remarkable that peroxysulfenic Cys145 (Cys145^peroxy^) is clearly detected in the SARS-CoV-2 3CL M^pro^ crystal instead of the sulfenic (—S—OH) or sulfinic [—S=O(OH)] acid cysteine modifications [Fig. 2 ▸(*a*)], which are far more prevalent thiol oxidation states (Rudyk & Eaton, 2014 ▸; Poole *et al.*, 2020 ▸); Peroxysulfenic cysteine has previously been observed in structures of cysteine dioxygenase containing a mononuclear nonheme iron, where this thiol oxidation state is presumably stabilized by a direct interaction of the Cys^peroxy^ distal-to-sulfur O atom with the iron cation (Simmons *et al.*, 2008 ▸; Driggers *et al.*, 2013 ▸). In structure **I** the O—O moiety of Cys145^peroxy^ is rotated into the oxyanion hole formed by the Ser139–Cys145 turn and forms hydrogen bonds to the main-chain amide N atoms of Gly143, Ser144 and Cys145^peroxy^ [Fig. 2 ▸(*b*) and Supplementary Fig. S2]. During catalysis, the oxyanion hole (Taranto *et al.*, 2008 ▸; Wu *et al.*, 2013 ▸) stabilizes the negative charge on the scissile peptide-bond carbonyl O atom when the covalent tetrahedral intermediate forms (Ménard & Storer, 1992 ▸; Tong, 2002 ▸; Rauwerdink & Kazlauskas, 2015 ▸). It can also stabilize the negatively charged O atom of some aldehyde inhibitors when the reactive aldehyde group forms a covalent C—S bond with Cys145, as was found in some structures of SARS-CoV 3CL M^pro^ (Yang *et al.*, 2003 ▸; Zhu *et al.*, 2011 ▸). However, in a recent structure of SARS-CoV-2 3CL M^pro^ in complex with an aldehyde inhibitor the aldehyde O atom faces in the opposite direction from the oxyanion hole to make a hydrogen bond to His41 (Dai *et al.*, 2020 ▸). Because hydrolase enzymes have an oxyanion hole that is specifically designed to interact with and stabilize the negatively charged O atom (Ménard & Storer, 1992 ▸; Tong, 2002 ▸; Rauwerdink & Kazlauskas, 2015 ▸), we predict that in the Cys145^peroxy^ moiety observed in our room-temperature structure the distal-to-sulfur O atom is likely to be deproton­ated and negatively charged (—S—O—O^–^).

### 3.2. Cys156 reacts with *N*-ethylmaleimide when Cys145 is oxidized at pH 7   

**Figure 3:**
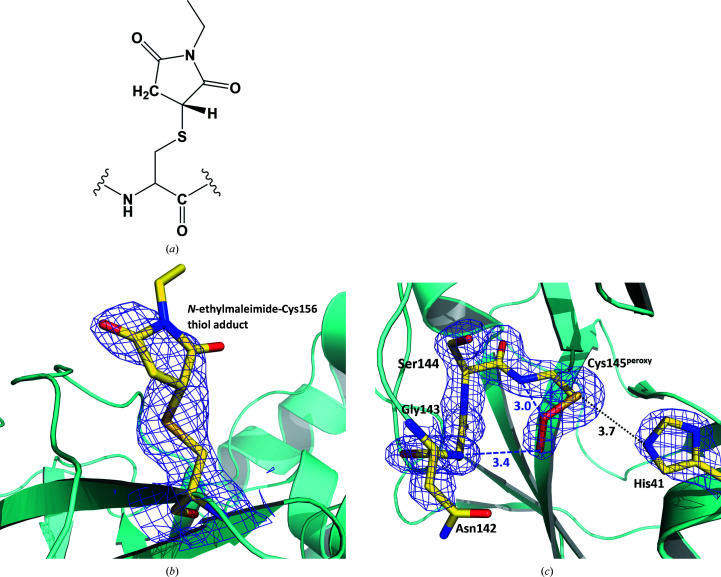
(*a*) A chemical diagram of an *N*-ethylmaleimide-conjugated cysteine residue. (*b*) The 2*F*_o_ − *F*_c_ electron-density map contoured at the 0.8σ level (purple mesh) for *N*-ethylmaleimide-conjugated Cys156 in structure **II**. (*c*) The catalytic site in structure **II**. Possible hydrogen bonds are shown as blue dashed lines; the distance between Cys145 and His41 is shown as a black dotted line. The 2*F*_o_ − *F*_c_ electron-density map contoured at the 1.5σ level is shown as a purple mesh. All distances are given in ångströms.

As Cys145 is the catalytic residue, the high reactivity of Cys145 towards molecular oxygen is to be expected. Interestingly, the conditions mentioned above, which resulted in the oxidation of Cys145, did not cause the oxidation of the other surface cysteine residues (Cys85, Cys156, Cys160 and Cys300) that are exposed to bulk solvent. The rest of the cysteine residues in 3CL M^pro^ reside in hydrophobic pockets that are shielded from the bulk solvent and are not expected to be reactive. To probe the reactivity of the surface cysteines, we manipulated another crystal in the same fashion as the one that gave structure **I** to make sure that Cys145 is oxidized and then soaked the crystal in a solution containing *N*-ethyl­maleimide (see Section 2). *N*-Ethylmaleimide is a known Michael reagent for the determination of reactive thiols in proteins (Hill *et al.*, 2009 ▸). We obtained a 1.80 Å resolution room-temperature X-ray structure from this crystal (Supplementary Fig. S1), which we call structure **II**. In this structure, we observed a product of *N*-ethylmaleimide Michael addition to Cys156 [Figs. 3 ▸(*a*) and 3 ▸(*b*) and Supplementary Fig. S2] with a refined occupancy of 80%, but no other exposed cysteines reacted with this alkylating reagent. Cys145 was again found in the peroxysulfenate oxidation state, identical to that in structure **I** [Fig. 3 ▸(*c*)], but its conformation was slightly different. In structure **II**, the distal-to-sulfur O atom in Cys145^peroxy^ rotates away from the oxyanion hole. It no longer makes reasonable hydrogen bonds to the main-chain amide N atoms of the Gly143, Ser144 and Cys145^peroxy^ residues. The closest contact is with NH of Gly143 at a distance of 3.4 Å, suggesting that this O atom might be protonated in structure **II**, and the Cys145^peroxy^ moiety is neutral (—S—O—OH).

### 3.3. The reduced thiols of Cys145 and Cys156 react with *N*-ethylmaleimide at pH 7   

**Figure 4:**
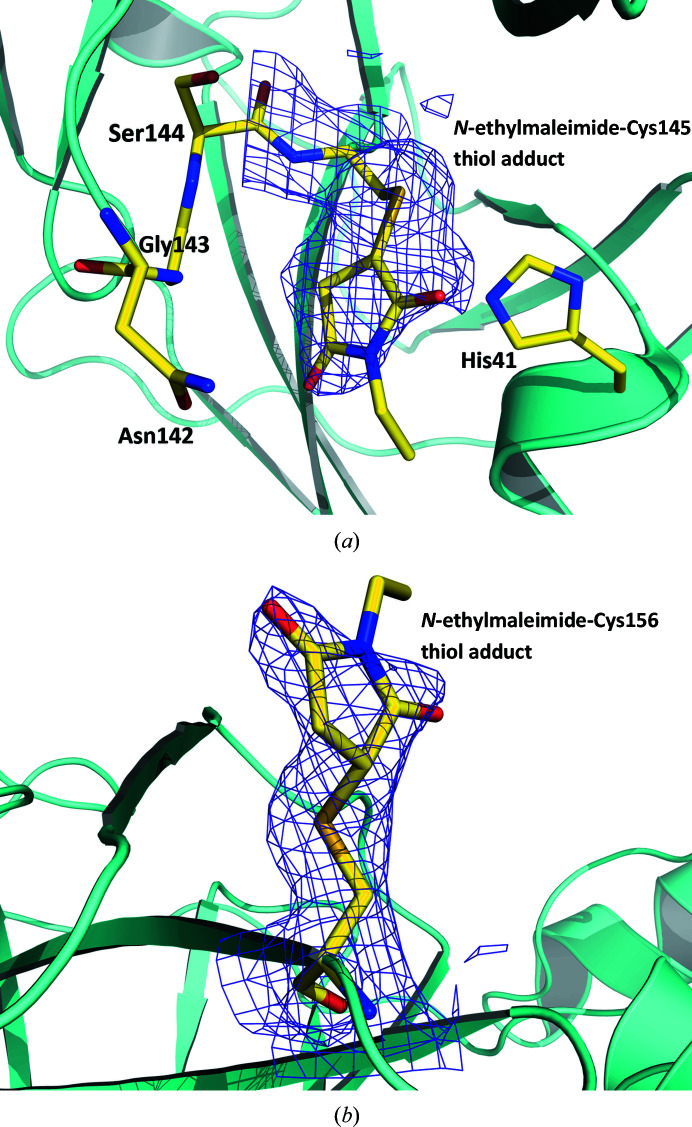
The 2*F*_o_ − *F*_c_ electron-density maps contoured at the 0.8σ level (purple mesh) for *N*-ethylmaleimide-conjugated Cys145 (*a*) and Cys156 (*b*) observed in structure **III**.

To confirm the reactivity of Cys145 towards *N*-ethylmale­imide and to eliminate the possibility that the lack of reactivity of other surface cysteine residues towards this alkylating agent as observed in structure **II** was owing to the presence of the oxidized catalytic Cys145, we soaked a fresh native crystal of SARS-CoV-2 3CL M^pro^ in a solution containing *N*-ethyl­maleimide (see Section 2 and Supplementary Fig. S1). Removing the exposure of the crystal to a potentially oxidizing environment produced structure **III**, in which we observed that both Cys145 and Cys156 are conjugated with *N*-ethylmaleimide (Fig. 4 ▸ and Supplementary Fig. S2), with refined occupancies of 76% and 78%, respectively; yet again, no other cysteines reacted. This result indicates that Cys85, Cys160 and Cys300 do not react with *N*-ethylmaleimide because they cannot be reached by this chemical owing to the crystal packing.

### 3.4. The Cys145 thiol remains reduced at pH 6   

To understand how the pH influences the reactivity of Cys145 towards oxidation, we grew crystals of SARS-CoV-2 3CL M^pro^ at pH 6 by dialyzing the enzyme in 20 m*M* bis-Tris pH 6 buffer and using a crystallization well solution with the same buffer and pH (0.1 *M* bis-Tris pH 6) (Supplementary Fig. S1). The crystals grew in a very similar unit cell to those of structures **I**–**III**; however, the space group changed from *I*2 for the pH 7 crystals to *P*2_1_ for the pH 6 crystals (Supplementary Table S1). In the *P*2_1_ structure **IV** there are two independent molecules of 3CL M^pro^ in the asymmetric unit forming the dimer. Careful examination of the electron-density maps for structure **IV** revealed that the Cys145 residues in both monomers are not oxidized, remaining in the reduced thiol state even after six weeks of crystal growth (Supplementary Fig. S3). At pH 6 the thiol of Cys145 is expected to be fully protonated and less reactive than the deprotonated thiolate. Protonation of Cys145 can thus explain why we observe it in the reduced state in structure **IV**.

We compared structure **IV** with the previously published pH 6 structure of 3CL M^pro^ from SARS-CoV, which was obtained in the same space group *P*2_1_ (PDB entry 1uj1; Yang *et al.*, 2003 ▸; Tan *et al.*, 2005 ▸). In PDB entry 1uj1, the S1 substrate-binding subsites have significantly different conformations in the two crystallographically independent monomers. In comparison with structure **IV** (Supplementary Fig. S4), the loop containing residues 138–140 showed dramatic conformational rearrangements owing to the reorientation of Glu166, resulting in the apparent collapse of the S1 subsite and the oxyanion hole. In our structure **IV**, however, we did not detect significant conformational differences within the substrate-binding site between the two monomers or when compared with our room-temperature structure of the native enzyme (Kneller *et al.*, 2020 ▸). It is possible that in SARS-CoV-2 3CL M^pro^ such S1 subsite collapse cannot occur, or this might be a structural artifact owing to the cryocooling of the SARS-CoV 3CL M^pro^ crystals.

## 4. Discussion   

It is important to consider what information can be deduced from our observations of the reactivity of Cys145 in SARS-CoV-2 3CL M^pro^ at different pH values and what insights can be gained regarding the catalytic mechanism of the enzyme. The experimental p*K*_a_ values for Cys145 and His41 were previously determined to be 8.0 ± 0.3 and 6.3 ± 0.1, respectively, for SARS-CoV 3CL M^pro^, which is 96% homologous to the SARS-CoV-2 enzyme (Huang *et al.*, 2004 ▸; Solowiej *et al.*, 2008 ▸). It can be anticipated that the p*K*_a_ values for the Cys–His dyad in the SARS-CoV-2 enzyme are similar. Therefore, at a pH of 7.0 most of the catalytic sites should contain a protonated Cys145 and a neutral His41; thus, Cys145 should not be particularly reactive. This is consistent with a previous suggestion that the ligand-free enzyme adopts the catalytically resting (nonreactive) state and is activated by proton transfer from the Cys145 thiol to the His41 imidazole upon substrate binding, generating a zwitterionic pair with a highly reactive Cys145 thiolate (Paasche *et al.*, 2014 ▸). However, theoretical quantum mechanics/molecular mechanics calculations using approximated coupled-cluster approaches have shown that this proton transfer has a high energy barrier of ∼24 kcal mol^−1^ (Paasche *et al.*, 2013 ▸), which is too high considering the measured *k*_cat_ of ∼1–8 s^−1^ (Solowiej *et al.*, 2008 ▸; Kuo *et al.*, 2004 ▸). Conversely, at the pH of 7.0 in our crystallization conditions, around 10–15% of the Cys145–His41 dyad side chains would be in the opposite protonation states, *i.e.* with a deprotonated negatively charged Cys145 and a protonated positively charged His41. It follows that these catalytic sites would be much more reactive and can be prone to oxidation; then, once all of them have reacted with oxygen dissolved in the solution, the rest of the molecules would re-equilibrate, generating more reactive deprotonated Cys145 species. At the physiological pH of 7.4, there would be close to 50% of deprotonated Cys145 residues.

Therefore, we suggest that the reactive species of the 3CL M^pro^ enzyme are those that already contain the Cys–His zwitterionic pair before a substrate (or covalent inhibitor) binds. Notably, our suggestion is in agreement with the normal solvent kinetic isotope effect (SKIE) on the burst rate in pre-steady-state studies and the inverse SKIE in steady-state measurements previously performed for SARS-CoV 3CL M^pro^. In contrast, these results are not consistent with rate-limiting general acid–base proton transfer from Cys145 to His41 (Solowiej *et al.*, 2008 ▸). When the pH of the crystallization solution was lowered to 6.0, we observed no oxidation of Cys145, indicating that the protonated thiol is not reactive towards oxygen, as expected. We also did not detect structural changes in the substrate-binding subsite S1 in our structure at pH 6.0, which was previously suggested to be the pH-triggered activation switch and to which the diminished activity of the SARS-CoV enzyme at acidic pH was attributed (Yang *et al.*, 2003 ▸; Tan *et al.*, 2005 ▸). Our study instead suggests that if the pH-dependence of SARS-CoV-2 3CL M^pro^ activity follows the same trend as that of the SARS-CoV enzyme, as should be expected, the enzyme activity must be governed by the protonation and deprotonation of the Cys–His dyad rather than by conformational changes of the active-site cavity.

If our hypothesis is correct, structure-based drug-design efforts should consider the reactive species of SARS-CoV-2 3CL M^pro^ in the design of covalent and noncovalent inhibitors. Such inhibitors may need to specifically target the enzyme active site with a negatively charged Cys145 and a positively charged His41 that create a different electrostatic environment compared with the neutral Cys–His dyad. Another important insight provided by our observations relates to whether the reactivity of Cys145 may be directly exploited for the design of novel inhibitors targeting SARS-CoV-2 3CL M^pro^. One possibility is to consider direct Cys145 oxidation by inhibitors containing an ozonide (or peroxide) reactive group (Dussault *et al.*, 1999 ▸), called a warhead, instead of the commonly utilized aldehyde, ketone or Michael acceptor warheads (Dai *et al.*, 2020 ▸; Zhang *et al.*, 2020 ▸; Jin, Du *et al.*, 2020 ▸; Pillaiyar *et al.*, 2016 ▸). Such inhibitors have been proposed as potential therapeutics against human cytomegalo­virus (Wang *et al.*, 2018 ▸). For example, a 1,2,4-trioxolane warhead (Dussault *et al.*, 1999 ▸) can directly oxidize the Cys145 thiol to a sulfenic acid, while generating an aldehyde ‘byproduct’, thus ultimately inactivating two 3CL M^pro^ molecules with one inhibitor molecule.

## 5. Conclusions   

We succeeded in trapping the SARS-CoV-2 3CL M^pro^ enzyme with Cys145 oxidized to the peroxysulfenic acid in the crystal and performed X-ray crystallographic experiments at a physiologically relevant temperature. We observed that none of the other solvent-exposed surface cysteine residues were oxidized. The catalytic Cys145 and surface Cys156 readily react with the alkylating reagent *N*-ethylmaleimide. Our structures indicate that Cys145 is highly reactive at physio­logical pH and suggest that the zwitterionic Cys145^−^–His41^+^ catalytic dyad is responsible for the initiation of catalysis, rather than proton transfer from Cys145 to His41 via a general acid–base mechanism upon substrate binding. At the acidic pH of 6.0, Cys145, as well as all other cysteines, appears to be nonreactive towards oxidation owing to the full protonation of its thiol. Our structures also point to possible improvements in the design of specific SARS-CoV-2 3CL M^pro^ inhibitors.

## Supplementary Material

PDB reference: SARS-CoV-2 3CL M^pro^, peroxysulfenic Cys145 structure (structure **I**), 6xb0

PDB reference: peroxysulfenic Cys145/*N*-ethylmaleimide Michael addition Cys156 structure (structure **II**), 6xb1

PDB reference: Cys145/Cys156 *N*-ethylmaleimide Michael addition structure (structure **III**), 6xb2

PDB reference: native structure at pH 6.0 (structure **IV**), 6xhu

Supplementary Table S1 and Figures S1-S4. DOI: 10.1107/S2052252520012634/mf5048sup1.pdf
