# Supplementary material for: Room-temperature X-ray crystallography reveals the oxidation and reactivity of cysteine residues in SARS-CoV-2 3CL Mpro: insights into enzyme mechanism and drug design
Source: IUCrJ. 2020 Sep 21;7(Pt 6):1028–35. doi: 10.1107/S2052252520012634 (PMC7553146; doi:10.1107/S2052252520012634)
Supplement: Supplementary file 1 [file m-07-01028-sup1.pdf]

IUCrJ

Volume 7 (2020)

Supporting information for article:

Room-temperature X-ray crystallography reveals the oxidation and reactivity of cysteine residues in SARS-CoV-2 3CL M^{pro}: insights into enzyme mechanism and drug design

Daniel W. Kneller, Gwyndalyn Phillips, Hugh M. O'Neill, Kemin Tan, Andrzej Joachimiak, Leighton Coates and Andrey Kovalevsky

Table S1 Data reduction and refinement statistics for the room temperature structures **I**, **II**, **III, IV** of the 3CL Mpro from SARS-CoV2.

Values in parentheses are for highest-resolution shell.

	**Structure I** (293K)<br>PDB ID 6XB0	**Structure II** (293K)<br>PDB ID 6XB1	**Structure III** (293K)<br>PDB ID 6XB2	**Structure IV** (293K)<br>PDB ID 6XHU
**Data collection:**	**X-ray (in-house)**	**X-ray (in-house)**	**X-ray (in-house)**	**X-ray (in-house)**
Diffractometer	Rigaku HighFlux Eiger 4M	Rigaku HighFlux Eiger 4M	Rigaku HighFlux Eiger 4M	Rigaku HighFlux Eiger 4M
Space group	I2	I2	I2	$P2_1$
Wavelength (Å)	1.5406	1.5406	1.5406	1.5406
Cell dimensions:				
a, *b*, *c* (Å)	44.98, 53.71, 113.25	44.85, 53.88, 113.52	45.28, 54.64, 115.18	45.28, 54.91, 114.94
a, b, g (°)	90, 100.27, 90	90, 100.25, 90	90, 100.70, 90	90, 101.22, 90
Resolution (Å)	55.68-1.80 (1.87-1.80)	55.87-1.80 (1.87-1.80)	56.57-2.10 (2.18-2.10)	28.18-1.80 (1.84-1.80)
No. reflections unique	23977 (2217)	23520 (2095)	16266 (1630)	51125 (2867)
R_{merge}	0.071 (0.502)	0.049 (0.347)	0.065 (0.598)	0.051 (0.802)
R_{pim}	0.040 (0.386)	0.030 (0.288)	0.038 (0.415)	0.026 (0.612)
$CC_{1/2}$	0.991 (0.610)	0.989 (0.795)	0.996 (0.483)	0.985 (0.615)
$I / \sigma I$	18.6 (1.14)	21.3 (1.49)	13.7 (1.09)	6.9 (1.30)
Completeness (%)	96.9 (89.5)	94.8 (84.4)	99.8 (99.9)	99.2 (94.5)
Redundancy	3.7 (2.5)	3.1 (2.1)	3.8 (2.9)	4.0 (2.4)
**Refinement:**				
Resolution				
R_{work} / R_{free}	0.1545/0.2010	0.1587/0.2019	0.1956/0.2572	0.2021/0.2464
Ramachandran statistics				
Favored (%)	97.67	97.34	96.71	97.53
Allowed (%)	2.33	2.66	3.29	2.47
Outliers (%)	0	0	0	0
R.M.S. deviations				
Bond lengths (Å)	0.008	0.009	0.008	0.011
Bond angles (°)	0.861	1.090	0.918	1.275
All atom clashscore	3.30	3.07	7.65	3.50

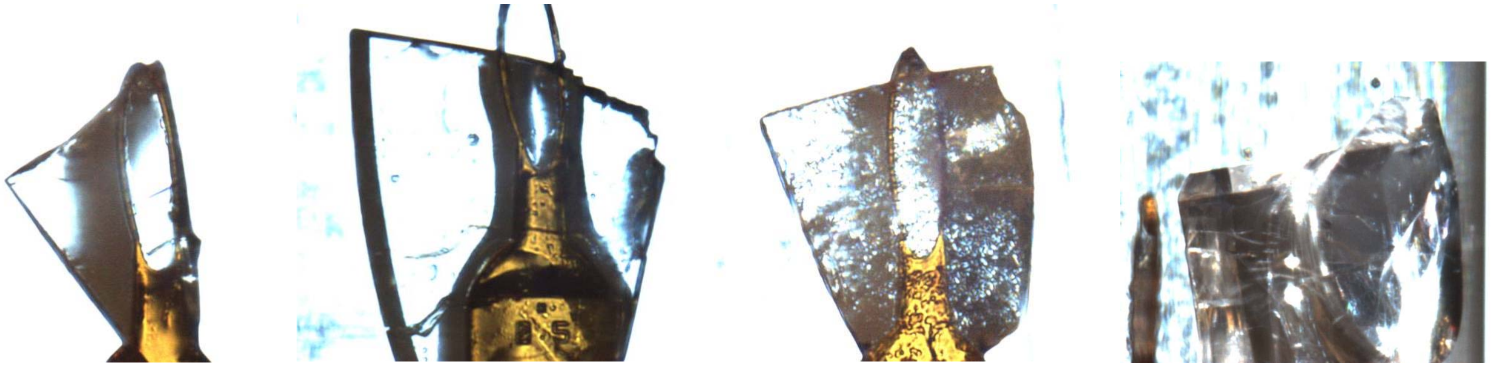

A **B** **C** **D**

Figure S1 Crystals of 3CL M^{pro} mounted on loops and used for the room-temperature X-ray data collection to give structures **I** (**A**), **II** (**B**), **III** (**C**) and **IV** (**D**).

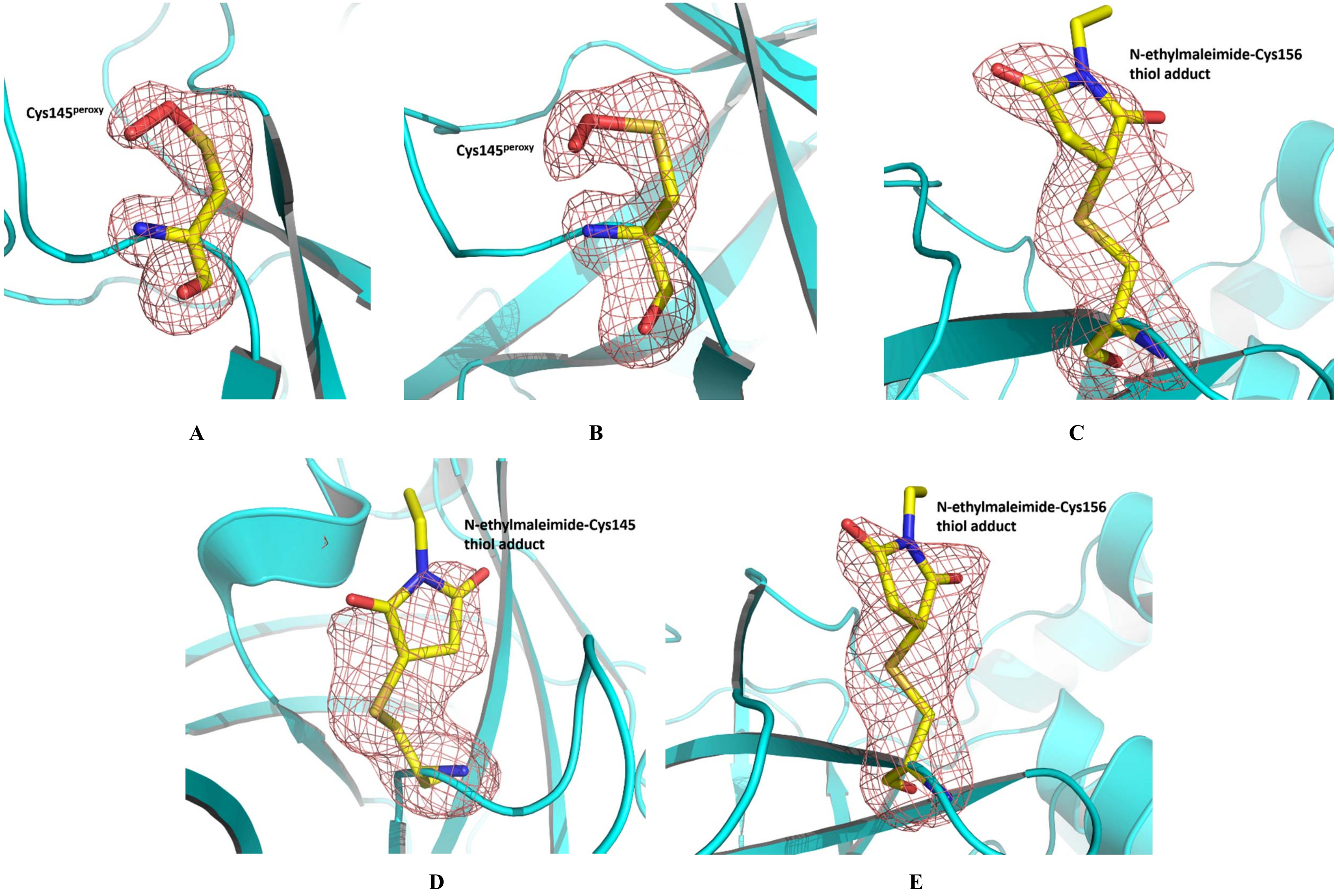

Figure S2 Polder omit F_O-F_C difference electron density maps of (**A**) Cys145^{peroxy} in structure **I** contoured at 4.0 σ level, (**B**) Cys145^{peroxy} in structure **II** contoured at 4.0 σ level, (**C**) N-ethylmaleimide-conjugated Cys156 in structure **II** contoured at 3.0 σ level; (**D**) N-ethylmaleimide-conjugated Cys145 in structure **III** contoured at 3.0 σ level; (**E**) N-ethylmaleimide-conjugated Cys156 in structure **III** contoured at 3.0 σ level

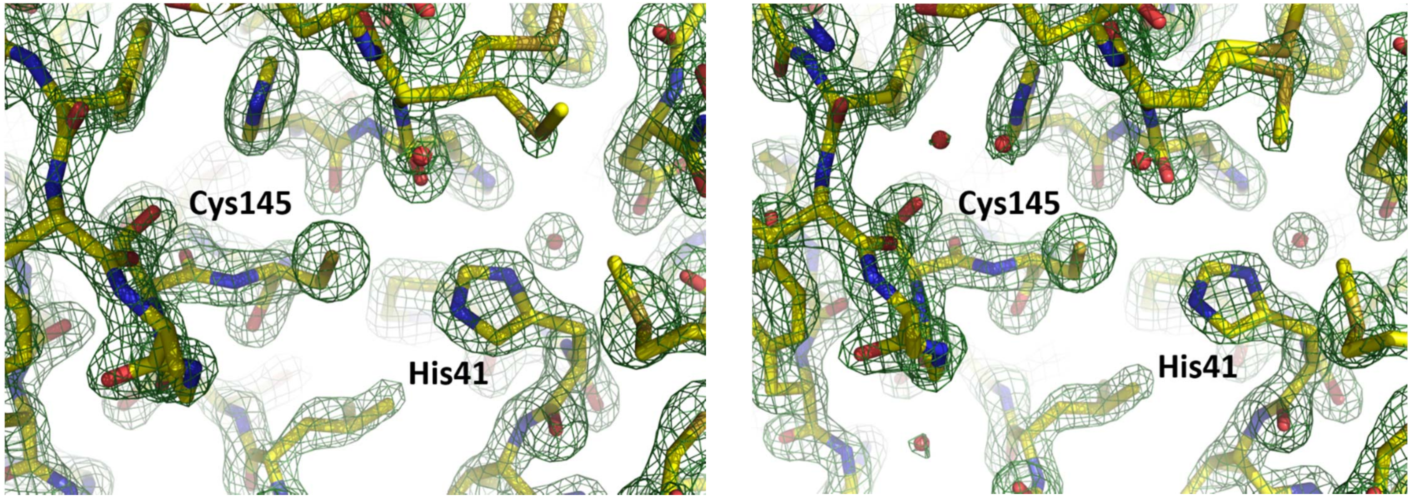

A **B**

Figure S3 (**A, B**) The $2F_O$-F_C electron density map contoured at 1.8 σ level (dark green mesh) for the catalytic sites and the surrounding residues observed in structure **IV** for the two crystallographically independent monomers.

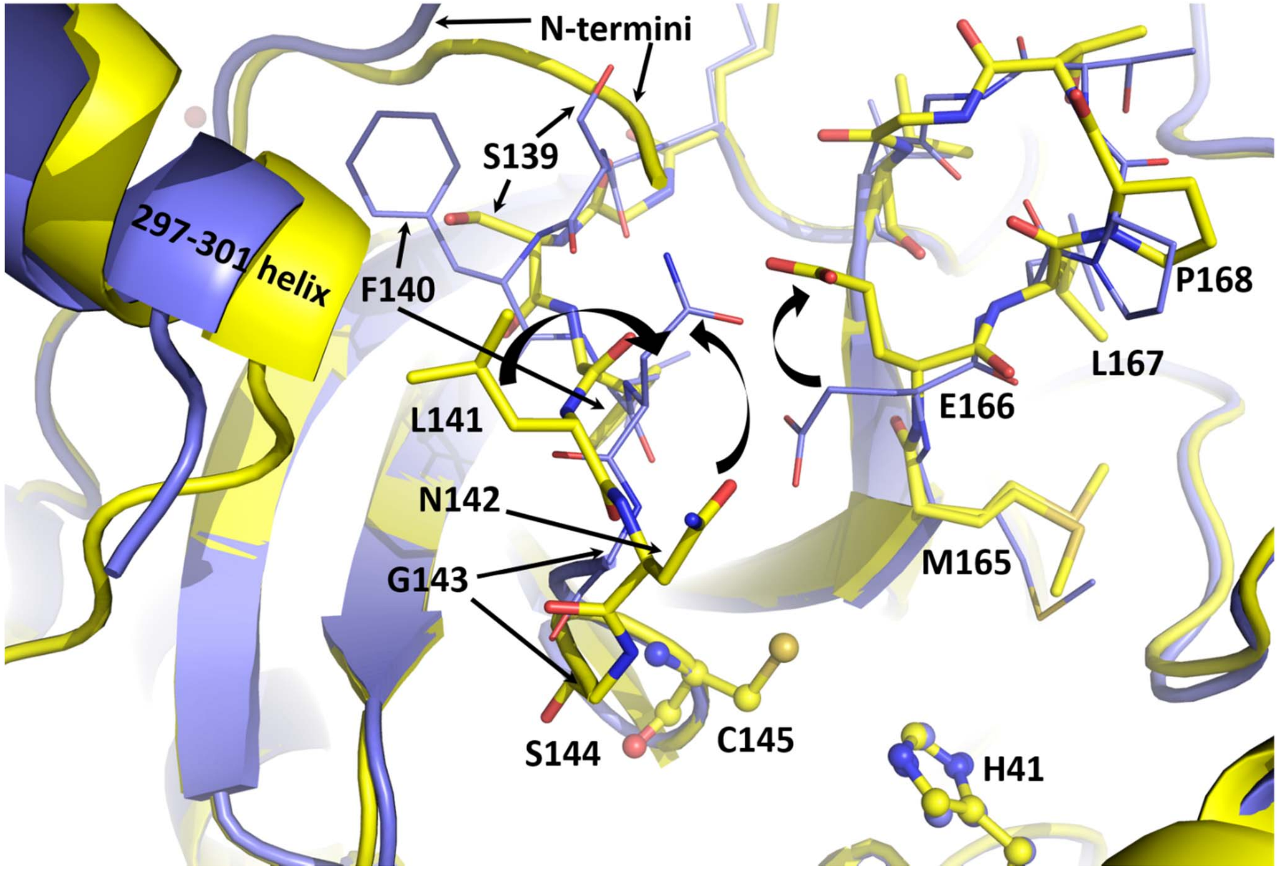

Figure S4 Superposition of the room-temperature $P2_1$ structure of SARS-CoV-2 3CL M^{pro} (yellow carbons) determined at pH 6 and the 100K $P2_1$ structure of SARS-CoV 3CL M^{pro} (purple carbons, PDB ID 1UJ1) at pH 6. In the latter structure a dramatic reorganization of the S1 substrate binding subsite results in the collapse of the oxyanion hole and removal of the N-terminus.
